# Dietary vitamin D_3_ supplementation protects laying hens against lipopolysaccharide-induced immunological stress

**DOI:** 10.1186/s12986-018-0293-8

**Published:** 2018-08-10

**Authors:** Yanqiang Geng, Qiugang Ma, Zhong Wang, Yuming Guo

**Affiliations:** State Key Lab of Animal Nutrition, College of Animal Science and Technology, China Agricultural University, Beijing, 100193 People’s Republic of China

**Keywords:** Vitamin D_3_, *Escherichia coli* lipopolysaccharide, Laying hens, Immunomodulation, Serum hormone

## Abstract

**Background:**

The effects of vitamin D on the immune function of laying hens are not well understood. This study investigated the effects of vitamin D_3_ (VD_3_) on laying performance and immunological functions in laying hens under *Escherichia coli* lipopolysaccharide (LPS) challenge.

**Methods:**

In experiment one, 360 Jinghong-1 strain layers (32 weeks) were randomly divided into four groups with six replicates per group and 15 hens per replicate. Hens were fed a basal diet supplemented with different levels of VD_3_ (0; 500; 1500; or 3000 IU VD_3_/kg of diet) for 10 weeks to determine laying performance, egg quality, and other parameters. In experiment two, 24 Jinghong laying hens (32 weeks) were fed basal diets with either 0 or 3000 IU VD_3_/kg of diet. After 10 weeks of feeding, six hens from each treatment were injected intravenously with 8 mg/kg of body weight of either LPS or saline. Blood and spleen samples were obtained for immune parameter analysis 4 h after injection.

**Results:**

VD_3_ deficiency reduced egg production and egg quality; in addition, feed intake and feed-to-egg ratio increased. No significant differences were observed in these parameters except eggshell strength between dietary VD_3_ supplemental levels at 500; 1500; and 3000 IU VD_3_/kg of diet. VD_3_ deficiency increased serum hormone (calcitonin, parathyroid hormone, estradiol, and progesterone) and cytokine (IL-6, IL-10) levels, the ratio of IFN-γ to IL-4, myeloperoxidase activity and total IgG content in the serum, and upregulated the blood CD3^+^ T cell population. Splenic retinoid X receptor (RXR), nuclear factor-κB (NF-κB), inducible nitric oxide synthase (iNOS), and polymeric immunoglobulin receptor (pIgR) gene mRNA levels were upregulated in VD_3_-deficienct hens. VD_3_ deficiency significantly reduced serum Follicle stimulating hormone (FSH) and Luteinizing hormone (LH) concentrations and the number of CD4^+^CD25^+^ T cells in the blood. These changes were completely normalized by VD_3_ sufficiency. LPS reduced serum LH concentration, splenic lysozyme, and pIgR gene mRNA levels. LPS induced an increase in total serum IgM levels and the percentage of CD8^+^ T cells in the blood. The changes were completely reversed by VD_3_ addition.

**Conclusion:**

VD_3_ supplementation could protect laying hens not only from VD_3_ deficiency but also from immunological stress.

## Background

Vitamin D_3_ (VD_3_), also known as cholecalciferol, is the inactive form of vitamin D that can be ingested through dietary intake or be generated endogenously in the skin of animals exposed to UV light. VD_3_ is converted into its active form, 1, 25-dihydroxycholecalciferol (1,25-(OH)_2_D_3_), following a two-step hydroxylation process mediated by two key enzymes, 25-hydroxylase and 1α-hydroxylase. The first hydroxylation step occurs in the liver by 25-hydroxylase, which hydroxylates cholecalciferol at the 25-C position to form 25-hydroxycholecalciferol (25(OH)D). The subsequent hydroxylation of 25-hydroxycholecalciferol occurs at the 1-C position and is mediated by 1α-hydroxylase in the kidneys to produce 1,25-(OH)_2_D_3_, the active form of vitamin D [1, 2]. This active form of vitamin D is thought to act via binding to the nuclear vitamin D receptor protein (VDR). The VDR then binds to target DNA sequences as a heterodimer with the retinoid X receptor (RXR), recruiting a series of coactivators, inducing target gene expression [3, 4]. Vitamin D is an essential nutrient and plays an integral role in maintaining calcium and phosphorus homeostasis, skeletal health, and muscle development [5–7]. Interestingly, increasing evidence derived from research in murine and human systems has demonstrated that vitamin D plays an important role in maintaining normal immune function and crosstalk between the innate and adaptive immune systems by influencing vitamin D receptors and activating enzymes [8–10]. Furthermore, VD_3_ has been shown to possess immunomodulatory, antioxidant, anti-inflammatory, antibacterial, antiviral, anti-allergy and cancer prevention properties [11–13]. In mammals, VD_3_ insufficiency and deficiency result in not only dysfunction of the innate and adaptive immune systems but also promotes micro-inflammation, as well as an increased risk of viral or bacterial infections [14–17]. Clinical trials of inexpensive VD_3_ supplements at appropriate doses show the vitamin’s effectiveness in the prevention and control of the diseases and inflammation through enhancing innate immunity, inducing antimicrobial peptide synthesis and nitric oxide production, dampening excessive inflammation responses, or decreasing the production of pro-inflammatory cytokines. The vitamin acts directly on T lymphocytes or antigen presenting cells [7, 8, 18–20]. In birds, the role of VD_3_ in calcium and phosphorus metabolism is crucial for its well-documented involvement in bone development and eggshell formation in laying hens. However, the immunomodulatory, anti-inflammatory and anti-coccidia roles of VD_3_ or its metabolites have also been demonstrated in chickens [21–24] and chicken cells [23, 25]. In addition, Rodriguez-Lecompte et al. [26] reported that vitamin D or 25(OH)D, both have a robust immunomodulatory property with a more favorable helper T cell (Th2) response in broiler chickens. Aslam et al. (1998) [27] observed that vitamin D deficiency depresses the cellular immune responses in young broiler chicks. Therefore, research into the immunomodulatory, anti-inflammatory, and anti-infection roles of vitamin D continues to highlight novel opportunities for the promotion of animal and poultry health based on implementation of vitamin D supplemented nutritional regimens [28].

Laying hens reared under intensive commercial conditions are often threatened by the large amounts of pathogen-associated molecular patterns, such as lipopolysaccharides (LPS) derived from gram-negative bacteria and lipoteichoic acid derived from gram-positive bacteria. Inhalation of environmental gram-negative bacteria (their endotoxins in particular) has been suggested to be a major poultry health problem. Administration of LPS to chickens stimulates the immune system, activating the transcription of nuclear factor (NF)-κB, releasing acute phase proteins and proinflammatory cytokines, causing the expression of toll-like receptors 2 (TLR2) and 4 (TLR4), and modulation of antibody responses [29], besides causing clinical symptoms such as fever, anorexia, and decreased growth [29, 30].

Interest in using nutritional strategies to modulate the immune functions caused by LPS in chickens has increased in the last decade. Significant accumulated evidence from mammalian studies have demonstrated that supplementation with VD_3_ inhibits inflammatory cytokine production in LPS-induced acute lung injury [31] and alleviates LPS-induced renal oxidative stress by regulating oxidant and antioxidant enzyme genes [12]. Some reports have shown that VD_3_ or 25-hydroxycholecalciferol exhibit anti-inflammatory effects in broilers or chicken immune cells following LPS administration [21–23]. However, little information has been reported about the effects of dietary VD_3_ supplementation or deficiency on immune responses and serum biochemical indexes of lying hens challenged with LPS or the underlying mechanisms of action. Thus, our study was designed to investigate the effects of dietary VD_3_ supplementation and deficiency on egg performance, blood T lymphocyte subset percentages, serum biochemical constituents, serum natural antibodies and cytokines, and mRNA abundance of splenic immune-related genes in laying hens (32 to 42 weeks of age) subjected to acute *Escherichia coli* LPS challenge, and to explore how laying hens respond to those manipulations. Such investigations may provide evidence of whether dietary VD_3_ supplementation exerts dual roles in laying hens following immune challenge, by improving egg performance and the immune function.

## Methods

### Animal ethics statement

This study, which complied with institutional and national guidelines for the care and use of animals, was approved by the Committee of Animal Experiments of China Agricultural University. All efforts were made to minimize animal suffering.

### Animal model and experimental treatments

Two experiments were conducted separately in this study. Experiment one was designed to study the effects of dietary VD_3_ levels on egg production, egg quality, and serum 25(OH)D levels of laying hens. A total of 360 Jinghong laying hens (Beijing Yukou Poultry Co., Ltd., China) at 32 weeks of age with similar weight, production performance, and genetic background were randomly assigned to four groups with six replicates per group and 15 birds per replicate. Hens were fed a corn–soybean meal basal diet supplemented with different levels of VD_3_ (0; 500; 1500; or 3000 IU VD_3_/kg) of diet. The basal diet was formulated to satisfy the Chinese Feeding Standard of Chickens (NY/T33–2004) and nutrient requirements of laying hens consuming 110 g of feed per day. The basal composition of the diet is shown in Table 1. The VD_3_ requirement of laying hens according to the Chinese Feeding Standard of Chickens (2004) is 1600 IU/kg of diet; however, the amount of VD_3_ added for laying hens in practical production is 3000 IU/kg of diet. The analyzed VD_3_ content of the laying hen basal diet was 0.00 IU/kg of diet (from natural VD_3_ in feed ingredients); no synthetic VD_3_ was included in the basal diet. Hens were housed in an experimental layer farm and kept in three-layer complete ladder cages at three birds per cage (40 × 45 × 45 cm) equipped with water nipples. During the test period, the birds were fed three times each day and supplied with water ad libitum. Additionally, a lighting procedure of 16 h illumination and 8 h darkness was maintained throughout the experiment. The average temperature in the chicken house was 24.5 ± 0.5 °C. Feed consumption was recorded weekly throughout the entire experimental period. Egg number, total egg weight and broken eggs of each replicate were recorded daily and calculated as percentage hen-day egg production, average egg weight, daily egg mass. and broken egg rate. Egg mass was calculated as egg weight × egg production. Feed efficiency (feed conversion rate, FCR) was calculated as grams of feed consumed per gram of egg mass (egg production/100 × egg weight) produced. Mortalities and health status were visually observed and recorded daily throughout the entire experimental period.

**Table 1:** Composition and nutrient levels of the experimental vitamin D_3_-deficient diets

Ingredients (%)	Composition	Calculated nutrient levels^c^	Value
Corn (7.8% crude protein)	66.45	Metabolic energy(MJ /kg)	11.30
Soybean meal (43% crude protein)	22.80	Crude Protein(%)	15.52
Limestone	8.20	Calcicum(%)	3.60
calcium hydrophosphate	1.70	Available phosphorus(%)	0.39
Sodium chloride	0.30	Lysine(%)	0.75
DL-methionine (98%)	0.12	Methionine(%)	0.37
Choline chloride (50%)	0.10	Met+Cys(%)	0.68
Vitamin premix^a^	0.03	Tryptophane(%)	0.18
Trace elements premix^b^	0.30	Threonine(%)	0.57

^a^Provided per kilogram of diet: vitamin A, 6000 IU; vitamin E, 21 IU; vitamin E, 21 IU; vitamin K3, 4.2 mg; vitamin B1, 3 mg; vitamin B2, 10.2 mg; folic acid, 0.9 mg; pantothenic acid, 15 mg; niacin, 45 mg; vitamin B6, 5.4 mg; vitamin B12, 24 μg; biotin, 0.15 mg. VD_3_ added alone. Prepared with a small mixing machine after premix mixing. High performance liquid chromatography to detect the actual content, and the calculation of the value of the match before the feed preparation

^b^Mineral premix provided per kilogram of complete diet: copper, 6.8 mg; iron, 66 mg; zinc, 83 mg; manganese, 80 mg; iodine, 1 mg; Se 0.3 mg

^c^ Nutrient levels were calculated from data provided by Feed Database in China (2012)

Experiment two was designed to study the effects of VD_3_ supplementation on immune response and serum reproductive hormones in laying hens challenged with LPS. After 10 weeks of a feeding trial, 12 laying hens from the control (without VD_3_ supplementation) and 12 laying hens from the VD_3_-supplemented group (supplemented 3000 IU VD_3_/kg of diet) were randomly selected, and six hens from each dietary treatment were injected intravenously with either 8.0 mg/kg body weight of *E. coli* LPS (serotype O111:B4, Sigma-Aldrich Inc., St. Louis, MO, USA) diluted in sterile saline. Feed was removed from the birds after injection. Four hours after LPS or saline injection, blood samples were collected via wing venipuncture and divided into two aliquots (2 to 3 mL each); one 4-mL serum vacutainer tube and one 4-mL vacutainer tube coated with K_2_EDTA (BD BioSciences, San Jose, CA, USA). These blood samples were kept on ice during collection, whereas the blood samples for serum were clotted at room temperature during collection, centrifuged at 12,000×*g* for 5 min at 4 °C, and serum was recovered for biochemistry analysis. After the blood collection, all birds were killed by cervical dislocation and splenic samples were collected and frozen immediately with liquid nitrogen and stored at − 80 °C until further analysis.

### Flow cytometry analysis

The percentages of CD3^+^, CD4^+^, CD8^+^, and CD4^+^CD25^+^ cells among peripheral blood lymphocytes were analyzed by flow cytometry as described previously [32]. Briefly, the blood from K_2_EDTA vacutainer tubes was diluted 1:1 with 1 × PBS and held on ice. The blood suspensions were carefully layered into centrifuge tubes containing an equal volume of Ficoll to form a discrete layer above the Ficoll. The tubes were centrifuged at 220×g for 30 min at room temperature, and the mononuclear layers (buffy coats) were removed, transferred to different centrifuge tubes, and washed twice in 1 × PBS. Immediately, the cells were counted on a hemocytometer using the trypan blue exclusion assay (Sigma-Aldrich Inc. USA) and the cell suspensions were adjusted to 1 × 10^6^ viable cells per mL of 1 × PBS. The following monoclonal antibodies were used in immunofluorescence assays: mouse anti-chicken-CD3-SPRD-labeled, mouse anti-chicken-CD4-FITC-labeled, mouse anti-chicken-CD8-RPE-labeled (Southern Biotechnology Associates Inc., Birmingham, AL, USA) and mouse anti-chicken CD25-Alexa Fluro 647-labeled (AbD Serotec, USA). Negative isotype control IgG (mouse IgG1κ-SPRD, mouse IgG1κ-FITC, mouse IgG1κ-R-PE, and HuCAL Fab-dHLX-MH negative control-Alexa Fluro 647-labeled (AbD Serotec, USA) were diluted in PBS (pH 7.2). Using 96-well round-bottom plates, 100 μL of each cell suspension was plated in duplicate. Each of the fluorescein-labeled antibodies was added to the respective wells, and the stained cells were incubated for 30 min at 4 °C in the dark. The cells were washed twice in cold 1 × PBS and centrifuged for 30 min at 1800 g to remove any traces of unbound antibodies. They were then transferred to 5-mL polystyrene round-bottom tubes for analysis. A total of 10,000 cells per sample were conducted using a Coulter XL (Beckman Coulter Corp., Fullerton, CA, USA) at Xi-Yuan Traditional Chinese Medicine Hospital, Chinese Academy of Medicine Science, China. The percentages of CD3^+^ T, CD4^+^ T, CD8^+^ T and CD4^+^ CD25^+^ T cells in PBMC were subsequently calculated.

### Serum calcium and 25-hydroxycholecalciferol analysis

Serum total calcium content (mM) was measured with the UV-2000 visible spectrophotometer (Unico Instruments Co. Ltd., Shanghai, China) using commercial colorimetric assay kits (Nanjing Jiancheng Bioengineering Institute, Nanjing, China).

To determine serum 25-hydroxycholecalciferol (25(OH)D), serum samples were separated by centrifugation at 894×g for 15 min and frozen at − 20 °C. Ultra-high-performance liquid chromatography–tandem mass spectrometry was used to assess 25(OH)D as previously described [33]. A standard curve was obtained using dilutions of a 25(OH)D standard (Iso Sciences, USA).

### Serum hormone analysis

Corticosterone (CORT), calcitonin (CT), parathyroid hormone (PTH), estradiol (E2), follicle stimulating hormone (FSH), luteinizing hormone (LH), testosterone (T), and progesterone (PG) levels in the serum were determined with commercial radioimmunoassay (RIA) kits in accordance with the manufacturer’s instructions (Beijing North Institute of Biological Technology, Beijing, China).

### Serum complement and cytokine analysis

Serum myeloperoxidase (MPO), complement components (C3, C4, C5) were measured using a commercial kit (Nanjing Jiancheng Bioengineering Institute). Serum concentrations of interleukins (IL-1β, IL-2, IL-4, IL-6, IL-10), interferon-γ (IFN-γ), and tumor necrosis factor-α (TNF-α) were determined with commercially available chicken cytokine ELISA kits (Beijing North Institute of Biological Technology), according to the manufacturer’s protocol. In each assay, a control recombinant chicken cytokine was diluted over the recommended detection range to generate a standard curve, and the linearity was calculated using Microsoft Excel 2013(Microsoft Corporation, Redmond, WA, USA) to be *R*^2^ = 0.99. Sample concentrations were interpolated from the standard curve.

### Serum total IgG and IgM determination

Serum levels of total IgG and IgM were quantified with chicken IgG and IgM ELISA kits, respectively (Bethyl Laboratories, Inc., Montgomery, TX, USA) following the manufacturer’s procedure. The serum samples were diluted 1:125,000 for IgG determination or 1:10,000 for IgM determination. The plates were read via an ELISA plate reader (SepctraMax® i3x Platform, Molecular Devices, LLC, San Jose, CA, USA) at 450 nm, and serum antibody concentrations were calculated using Gen 5 software (BioTek Instruments Inc., Winooski, VT, USA).

### Quantitative real-time PCR for measuring immune-related gene transcript levels in the spleen

Total RNA was isolated from snap-frozen spleen tissue samples (50 mg) based on the RNeasy mini kit following the animal tissue protocol (Qiagen Sciences, Inc., Germantown, MD, USA). The purity and concentration of the total RNA were measured in a NanoDrop-2000 spectrophotometer (ThermoFisher Scientific Co., Waltham, MA, USA) using the 260:280 nm absorbance ratio. First-strand cDNA was synthesized from 2 μg of total RNA using a PrimeScript RT reagent kit with gDNA eraser (Perfect Real Time; Takara Biomedical Technology (Beijing) Co. Ltd., Beijing, China) according to the manufacturer’s instructions and stored at − 20 °C until further processing. Primer sequences for chicken β-actin, toll-like receptor (TLR)-4, TLR-2, TNFSF15, IL-1β, IL-6, IL-8, nuclear factor κB (NF-κB), polymeric immunoglobulin receptor (pIgR), VDR, RXR, interferon gamma (IFN-γ), lysozyme (LYZ), and inducible nitric oxide synthase (iNOS) (Table 2) were designed based upon sequences available from public databases using Primer Express, version 5.0 (Applied Biosystems, Foster City, CA, USA) and synthesized by Sangon Biotech (Shanghai) Co., Ltd., Shanghai, China. Primers were designed to span an intron to avoid genomic DNA amplification. Quantitative real-time PCR was performed using the 7500 Fast Real-Time PCR system (Applied Biosystems) and SYBR Premix Ex Taq kit (Takara Biotechnology Co. Ltd.). Reactions were conducted in a 20-μL reaction mixture containing 10.0 μL of SYBR Premix Ex Taq (2×) mix, 2.0 μL of cDNA, 0.5 μL of each primer (10 mmol/L), and 7.0 μL of sterile nuclease-free water. For PCR, samples were subjected to an initial denaturation phase at 95 °C for 5 min, followed by 40 cycles of denaturation at 95 °C for 30 s, and annealing and extension at 60 °C for 30 s. Melt-curve analysis was performed to confirm PCR amplification specificity. All tissue samples used in cDNA synthesis and in the following PCR amplifications were analyzed in triplicate. Gene expression levels of TLR4, TNFSF15, IL-1β, IL-6, IL-8, IFN-γ, LYZ, and iNOS were analyzed with β-actin (β-actin values were designated the VD3 sufficiency hens without LPS injection**)** as an endogenous control. The average gene expression of each sample relative to that of β-actin was calculated using the 2^−ΔΔCt^ method.

**Table 2:** Sequences of primers for quantitative real-time PCR

Target gene	Primer sequence 5′ → 3′	Product size (bp)	Annealing temperature (°C)	GenBank No.	Efficiency (%)
VDR	F: TGGGAAAGGCGATGCTGATG	169 bp	59.0	NM_205098.1	86.5
	R: GATGCGAGACATGCAGATG				
RXR	F: GATGCGAGACATGCAGATG	161 bp	55.0	XM_015279790.1	96.3
	R: CGGGGTATTTGTGCTTG				
TLR2	F: ACCTTCTGCACTCTGCCATT	131 bp	58.5	NM_204278.1	94.4
	R: TGTGAATGAAGCACCGGTAA				
TLR4	F: CCACTATTCGGTTGGTGGAC	86 bp	59.0	NM_001030693.1	98.1
	R: ACAGCTTCTCAGCAGGCAAT				
NF-κB	F: ACCCCTTCAATGTGCCAATG	274 bp	59.3	NM_205129.1	80.5
	R: TCAGCCCAGAAACGAACCTC				
TNF-α	F: CCCCTACCCTGTCCCACAA	67 bp	60.7	NM204267.1	107.9
	R: TGAGTACTGCGGAGGGTTCAT				
IFN-γ	F: AAAGCCGCACATCAAACACA	64 bp	58.8	NM_205149.1	90.4
	R: GCCATCAGGAAGGTTGTTTTTC				
IL-1β	F: CAGCAGCCTCAGCGAAGAG	86 bp	60.5	NM_204524.1	98.7
	R: CTGTGGTGTGCTCAGAATCCA				
IL-6	F: AGATGGTGATAAATCCTGATGA	150 bp	54.5	NM_204628.1	130.4
	R: CGGTTTTCTCCATAAATGAAGT				
IL-8	F: GGCTTGCTAGGGGAAATGA	200 bp	59.0	NM_205498.1	88.6
	R: AGCTGACTCTGACTAGGAAACTGT				
LYZ	F: GACGATGTGAGCTGGCAG	225 bp	57.1	NM_205281.1	98.4
	R: GGATGTTGCACAGGTTCC				
iNOS	F: GAACAGCCAGCTCATCCGATA	103 bp	59.8	NM_204961.1	78.1
	R: CCCAAGCTCAATGCACAACTT				
pIgR	F: ATGAAGCAGAGCCAGGAGAC	103 bp	59.7	NM_001044644.1	106.4
	R: GAGTAGGCGAGGTCAGCATC				
β-actin	F: GAGAAATTGTGCGTGACATCA	282 bp	58.0	NM 205518	97.8
	R: CCTGAACCTCTCATTGCCA				

*IL* interleukin; *iNOS* inducible nitric oxide synthase; *pIgR* polyglobulin receptor; *TLR* toll receptor; *TNF-α* tumor necrosis factor; *LYZ* lysozyme; *RXR* retinoid X receptor; *VDR* vitamin D receptor

### Statistical analysis

In experiment one, a replicate was used as the experimental unit. Data were subjected to one-way ANOVA using the GLM procedure of SPSS 17.0 for Windows (SPSS Inc., Chicago, IL, USA). The treatment means were separated by Duncan multiple range tests at *P <* 0.05 significance levels.

In experiment two, a completely randomized design with two dietary treatments and two levels of immunological challenge in a 2 × 2 factorial arrangement was used. A two-way ANOVA (the GLM procedure of SPSS 19.0 for Windows (IBM Corp., Armonk, NY, USA) was used to examine the interactive and main effects of LPS and dietary VD_3_ levels on the dependent variables. Interactions were removed when the observed *P* value for interaction was above 0.10. When interactions were significant (*P* <  0.05), differences between means were determined using Tukey’s procedure. Differences with an α level of *P* <  0.05 was considered to be statistically significant.

## Results

### Effects of VD_3_ deficiency and VD_3_ sufficiency on egg production and egg quality

The results of the analysis of the production characteristics data are shown in Table 3; hens fed diets without VD_3_supplementation showed a significant reduction (*P* <  0.05) in egg production, daily egg mass and average egg weight. There was a significant increase in daily feed intake, broken egg rates and feed conversion ratio (FCR), compared with the other three different levels of VD_3_ -supplemented treatments. While statistical analysis showed that no significant (*P* > 0.05) differences in the above parameters of laying hens between dietary VD_3_ supplemental levels at 500 IU; 1500 IU and 3000 IU/kg of diet.

**Table 3:** Effect of different levels of vitamin D_3_ on laying performance of laying hens*

Items	Dietary VD_3_ levels, IU/kg					
	0	500	1500	3000	SEM	*P*-value
Hen-day egg production (%)	27.95^b^	90.30^a^	94.03^a^	92.29^a^	5.93	< 0.001
Average egg weight(g)	58.39^b^	60.43^a^	60.44^a^	59.93^a^	0.26	0.007
Day feed intake (g /d /hen)	137.60^a^	113.76^b^	116.10^b^	113.88^b^	2.20	< 0.001
FCR (g feed / g egg)	9.56^a^	2.12^b^	2.07b	2.09^b^	0.74	< 0.001
Daily egg mass(g/hens/d)	16.30^b^	54.55^a^	56.83^a^	55.31^a^	3.62	< 0.001
Broken egg ratio (%)	5.48^a^	0.29^b^	0.17^b^	0.27^b^	0.01	< 0.001
Death ratio (%)	8.47	3.36	5.62	3.06	0.01	0.063

*Data are presented as mean ± SEM (*n* = 90 hens /group)

^a-c^Means within a row without a common superscript differ significantly (*P <* 0.05)

*FCR* g of feed / g of egg mass

Compared with VD_3_ the deficiency group, dietary VD_3_ supplementation significantly (*P* <  0.05) increased eggshell thickness and egg strength (*P* <  0.01), whereas it had no remarkable (*P* > 0.05) effect on Haugh unit, eggshell color, and yolk color (Table 4). Egg strength in laying hens with 1500 IU and 3000 IU/kg dietary VD_3_ was significantly greater than that of hens fed 500 IU VD_3_/kg of diet.

**Table 4:** Effects of different levels of vitamin D_3_ on egg quality of laying hens*

Items	Dietary VD_3_ levels, IU/kg					
	0	500	1500	3000	SEM	*P* -value
Eggshell thickness(mm)	0.35^b^	0.43^a^	0.44^a^	0.45^a^	0.01	< 0.001
Eggshell strength(kg/cm^2^)	1.89^c^	3.23^b^	3.66^a^	3.94^a^	0.13	< 0.001
Haugh unit	80.36	80.33	82.35	80.08	0.83	0.764
Eggshell color	9.43	9.79	10.00	10.07	0.14	0.386
Yolk color	6.71	6.36	7.00	6.14	0.20	0.444

*Data are presented as mean ± SEM (n = 90 hens /group)

^a-c^Means within a row without a common superscript differ significantly (*P <* 0.05)

### Serum calcium and 25-hydroxycholecalciferol levels

**Fig. 1:**
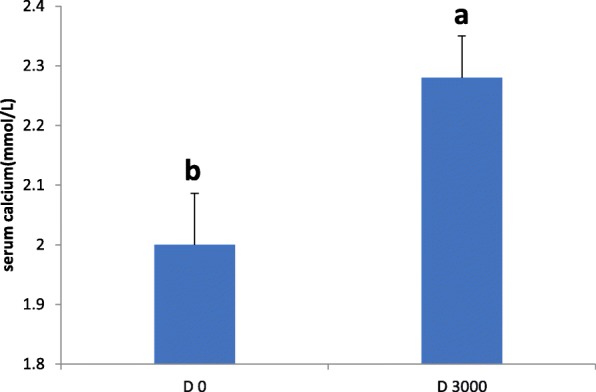
Serum calcium levels in vitamin D_3_-deficient and vitamin D_3_-sufficient laying hens. Data are presented as mean ± SEM (n = 6 /group). Bars with different letters (a, b) show a significant difference between VD3 deficiency and sufficiency (*P* < 0.05)

**Fig. 2:**
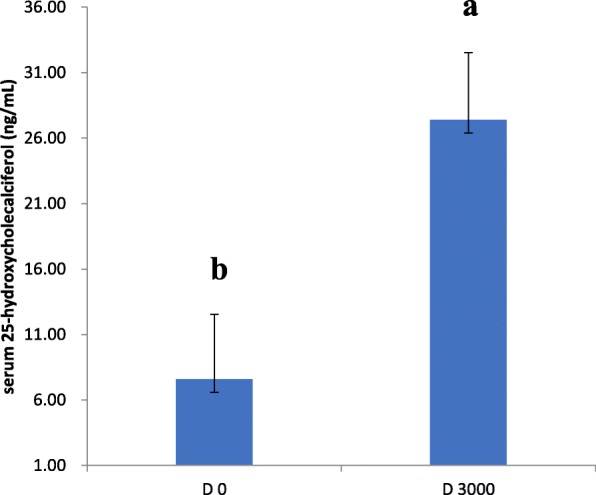
Serum 25-(OH)-D_3_ concentration in vitamin D_3_-deficient and vitamin D_3_-sufficient laying hens. Data are presented as mean ± SEM (n = 6 /group). Bars with different letters (a, b) show a significant difference between VD3 deficiency and sufficiency (P < 0.05)

There was a significant reduction in the levels of serum calcium (2.00 ± 0.98 mg/dL vs. 2.28 ± 1.17 mg/dL; *P* <  0. 05; Fig. 1) in the VD_3_-deficient group compared with the VD_3_-sufficient group. Furthermore, serum 25(OH)D levels were significantly lower in VD_3_-deficient birds compared with VD_3_-sufficient birds (7.60 ± 2.85 ng/mL vs. 27.39 ± 2.97 ng/mL; *P* <  0.05; Fig. 2).

### Serum hormone concentrations

The concentrations of hormones in serum showed (Table 5) that no significant diet × challenge interactions were found in the levels of serum CORT, CT, PTH, FSH, E2, T, and PG: LH was the exception. LPS-injected hens fed with the VD_3_-supplemented diets showed the highest levels of serum LH (*P* <  0.05) at 4 h after treatment with LPS compared with the other treated hens.

**Table 5:** Effect of vitamin D_3_ on serum biochemical and hormones of laying hens infected with LPS^*^

Items	Dietary VD_3_ levels, IU/kg					*P*-values		
	0		3000					
	Saline	LPS	Saline	LPS	SEM	VD_3_	LPS	Interaction
CORT (nmol/L)	78.79	91.37	76.78	82.78	5.00	0.169	0.036	0.295
CT (pg/ml)	222.50	191.98	182.17	171.34	5.61	0.001	0.016	0.216
PTH (ng/dl)	78.47	75.94	56.28	45.04	3.89	< 0.001	0.133	0.331
FSH (mIU/ml)	9.27	6.85	13.10	13.90	0.83	< 0.001	0.344	0.075
LH (pg/ml)	9.04^bc^	7.13^c^	10.36^b^	15.46^a^	0.82	< 0.001	0.041	< 0.001
E2 (pg/ml)	126.41	33.81	81.81	21.00	11.01	0.035	< 0.001	0.219
P (pg/ml)	2.36	1.32	1.86	0.69	0.17	0.002	< 0.001	0.693
T (ng/dl)	71.42	33.73	86.19	28.65	6.27	0.423	< 0.001	0.112

*Data are presented as mean ± SEM (*n* = 6 hens /group)

^a-c^ Data in the same row with no common superscript differ significantly (*P* <  0.05)

*SEM* standard error of the mean; *LPS* lipopolysaccharide; *CORT* cortisol; *CT* Calcitonin; *PTH* parathyroid hormone; *FSH* Follicle stimulating hormone; *E2* Estradiol; *LH* Luteinizing hormone; *T* testosterone; *P* progesterone

Relative to saline-injected hens, injection with LPS significantly reduced (*P* <  0.05) the concentrations of CT, E2, LH, T, and PG in the serum and significantly increased (*P* <  0.05) serum CORT concentration, whereas LPS had no significant effect (*P* > 0.05) on PTH and FSH. Compared with the VD_3_-deficient group, dietary VD_3_ supplementation significantly reduced (*P* <  0.05) the concentration of serum CT, PTH, E2, and PG, and significantly increased (*P* <  0.05) serum FSH and LH content, whereas LPS had no significant influence (*P* > 0.05) on serum CORT and T levels.

### Serum MPO activity

As shown in Table 6, LPS challenge resulted in lower MPO (*P* <  0.05) activity compared with unchallenged birds. However, relative to VD_3_ sufficiency, VD_3_ deficiency significantly (*P* <  0.05) increased serum MPO activity. Furthermore, there was a significant diet × LPS injection interaction for serum MPO activity and IgM concentration. LPS-injected hens fed with a VD_3_-supplemented diet showed a significant reduction (*P* <  0.05) in MPO activity compared with saline-injected hens without VD_3_ supplementation, whereas there were no significant differences (*P* > 0.05) compared with the other treatments.

**Table 6:** Effect of vitamin D_3_ on serum MPO, complement and natural antibody of laying hens infected with LPS^*^

Items	Dietary VD_3_ levels, IU/kg					*P-*values		
	0		3000					
	Saline	LPS	Saline	LPS	SEM	VD_3_	LPS	Interaction
MPO (ng/ml)	39.23^a^	21.04^b^	24.29^b^	22.62^b^	1.98	0.011	< 0.001	0.003
C3 (g/L)	0.87	0.78	0.88	0.76	0.03	0.896	0.054	0.751
C4 (g/L)	0.37	0.23	0.25	0.20	0.03	0.141	0.058	0.367
C5 (μg/ mL)	73.94	55.7	79.06	52.69	4.47	0.896	0.012	0.615
IgG (mg/mL)	21.4	17.96	18.76	15.25	1.17	0.038	0.009	0.977
IgM (μg/mL)	253.01^b^	252.67^b^	328.70^a^	163.04^c^	35.63	0.851	0.041	0.043

*Data are presented as mean ± SEM (n = 6 hens /group)

^a-c^Data in the same row with no common superscript differ significantly (*P* <  0.05)

*SEM* standard error of the mean; *LPS* lipopolysaccharide; *MPO* myeloperoxidase; *C3* complement 3; *C4* complement 4; *C5* complement 5; *IgG* immunoglobulins G; *IgM* immunoglobulins M

### Serum complement, IgG, and IgM concentrations

As shown in Table 6, LPS injection significantly decreased (*P* <  0.05) serum C5 activity, and total IgG and IgM concentration, compared with the saline-injected control. Total IgG was lower (*P* <  0.05) in the VD_3_-sufficient hens compared with the nonsupplemented hens. Furthermore, there was an interaction effect for total IgM concentration between LPS injection and VD_3_ treatment. Total IgM at 4 h after treatment with LPS was found to be lower (*P* <  0.05) in VD_3_-sufficient hens that were injected with LPS compared with the VD_3_-added hens that were not treated with LPS, whereas there was no significant difference (*P* > 0.05) compared with the VD_3_-deficient groups.

### T lymphocyte population in the blood

As shown in Table 7, compared to saline-injected hens, injection with LPS significantly reduced (*P* <  0.05) the levels of CD3^+^, CD4^+^ and CD8^+^ T cells in the blood, and significantly increased (*P* <  0.05) the percentage of CD4^+^CD25^+^ T cells following LPS challenge.

**Table 7:** Effect of vitamin D_3_ on peripheral blood mononuclear cell of laying hens infected with LPS^*^

Items	Dietary VD_3_ levels, IU/kg					*P*-values		
	0		3000					
	Saline	LPS	Saline	LPS	SEM	VD_3_	LPS	Interaction
CD3	73.68	37.02	63.30	23.02	4.96	0.042	< 0.001	0.750
CD4	55.17	27.13	46.13	17.73	3.91	0.087	< 0.001	0.972
CD8	9.80^a^	5.90^b^	10.20^a^	2.23^c^	0.79	0.080	< 0.001	0.033
CD4^+^CD25^+^	37.70	68.60	47.83	80.68	4.23	0.039	< 0.001	0.848
CD4^+^/CD8^+^	5.98	6.06	4.62	12.62	1.19	0.236	0.072	0.077

*Data are presented as mean ± SEM (n = 6 hens /group)

^a-c^ Data in the same row with no common superscript differ significantly (*P* < 0.05)

*SEM* standard error of the mean; *LPS* lipopolysaccharide

Sufficient supplementation with VD_3_ significantly decreased (*P* <  0.05) the percentage of CD3^+^ T lymphocytes and increased the population of CD4^+^CD25^+^ T cells (*P* <  0.05) in peripheral blood, compared with the nonsupplemented hens.

There was a significant interaction effect for the percentage of CD8^+^ T cells in the blood between dietary VD_3_ treatment and LPS challenge. LPS-injected hens fed diets with supplemental VD_3_ exhibited the lowest (*P* <  0.05) percentage of CD8^+^ T cells in the blood compared with the other three treatment groups.

### Serum cytokine levels

To determine whether VD_3_ could exert an anti-inflammatory effect in hens, we assessed the effect of VD_3_ on LPS-induced inflammatory response by measuring TNF-α, IFN-γ, IL-1β, IL-2, IL-4, IL-6, IL-8, and IL-10 concentrations in serum treated with LPS with or without VD_3_. Compared with the saline-injected control hens, LPS injection significantly (*P* <  0.05) increased the levels of TNF-α, IL-1β, IL-2, IL-4, IL-6 and IL-8 in the serum (Table 8). Supplementation of VD_3_ significantly (*P* <  0.05) reduced serum IL-6 and IL-10 amounts and the ratio of IFN-γ to IL-4 compared with the VD_3_-deficient grouping.

**Table 8:** Effect of vitamin D_3_ and LPS challenge on serum cytokines of laying hens infected with LPS^*^

Items	Dietary VD_3_ levels, IU/kg					*P*-values		
	0		3000					
	Saline	LPS	Saline	LPS	SEM	VD_3_	LPS	Interaction
TNF-α (pg/ml)	134.29	183.78	114.62	168.08	7.53	0.114	< 0.001	0.854
IFN-γ (pg/ml)	66.71	77.66	60.85	67.70	3.09	0.211	0.162	0.741
IL-1β (pg/ml)	15.01	18.65	13.15	17.26	0.60	0.085	< 0.001	0.792
IL-2 (pg/ml)	0.90	1.37	1.05	1.62	0.09	0.154	< 0.001	0.726
IL-4 (pg/ml)	25.95	49.70	24.50	48.36	3.39	0.777	< 0.001	0.992
IL-6 (pg/ml)	90.31	145.60	81.79	124.79	6.47	0.031	< 0.001	0.337
IL-8 (pg/ml)	28.49	48.34	25.96	44.06	2.74	0.400	< 0.001	0.828
IL-10 (pg/ml)	53.71	43.01	37.21	35.35	2.16	0.001	0.066	0.186
IFN-γ/IL-4	2.57	1.56	2.48	1.40	0.26	0.001	0.686	0.729

*Data are presented as mean ± SEM (n = 6 hens /group)

^a-c^ Data in the same row with no common superscript differ significantly (*P* < 0.05)

*SEM* standard error of the mean; *LPS* lipopolysaccharide; *TNF-α* tumor necrosis factor; *IL* interleukin; *IFN* interferon

### Splenic immune-related gene expression

The effects of dietary supplementation with VD_3_ on VDR, RXR, TLR-2, TLR-4, NF-κB, TNF-α, IFN-γ, IL-1β, IL-6, IL-8, LYZ, iNOS, and pIgR gene transcript abundance in the spleen of hens after LPS exposure are presented in Table 9. The expression levels of the TLR4, NF-κB, and IL-6 genes were remarkably increased (*P* <  0.05) by LPS exposure as compared with the unexposed control group. In the absence of LPS, the addition of VD_3_ remarkably (*P* <  0.05) prevented the upregulation of RXR-α, LYZ, iNOS, and pIgR genes expression in the spleen compared with VD_3_-deficient hens, while in the presence of LPS, supplemental VD_3_ upregulated (*P* <  0.05) splenic LYZ and pIgR gene mRNA levels but down-regulated (*P* <  0.05) the expressions of TLR4 and NF-κB gene in the spleen relative to the VD_3_-sufficient hens. There was a significant diet × challenge interaction for TLR2, pIgR, and LYZ expression in the spleen. LPS challenge significantly down-regulated (*P* <  0.05) splenic TLR2, LYZ, and pIgR gene mRNA levels, and markedly upregulated (*P* <  0.05) NF-κB gene mRNA levels, while these changes were normalized by VD_3_ supplementation. However, feeding with VD_3_ did not alter (*P* > 0.05) the expression levels of VDR, TLR-4, TNF-α, IL-1β, IL-6, and IL-8 in the spleen of hens compared with the nonsupplemented hens.

**Table 9:** Gene expression in the spleens of laying hens fed diets with or without supplemental dietary VD_3_^*^

Items	Dietary VD_3_ levels, IU/kg					*P-*values		
	0 IU/kg		3000 IU/kg					
	Saline	LPS	Saline	LPS	SEM	VD_3_	LPS	Interaction
VDR	1.52	1.22	1.16	1.71	0.14	0.998	0.629	0.200
RXR-α	1.96	1.78	1.14	1.05	0.14	0.020	0.824	0.344
TLR2	1.82^a^	0.76^b^	0.98^b^	1.04^b^	0.14	0.143	0.094	0.042
TLR4	1.13	3.17	1.22	1.97	0.24	0.300	0.020	0.620
NF-κB	1.56^b^	2.64^a^	1.08^b^	1.58^b^	0.18	0.009	0.011	0.029
TNF-α	0.98	1.25	1.12	1.74	0.11	0.130	0.061	0.815
IFN-γ	0.73	0.89	0.98	0.98	0.11	0.559	0.053	0.443
IL-1β	1.08	0.88	1.28	0.86	0.19	0.700	0.220	0.960
IL-6	0.51	2.30	0.81	1.94	0.23	0.893	< 0.001	0.439
IL-8	1.58	1.80	1.11	1.97	0.19	0.879	0.940	0.181
LYZ	1.84^a^	0.52^b^	0.47^b^	1.43^a^	0.20	0.561	0.453	0.003
iNOS	2.10	1.82	1.30	1.12	0.17	0.016	0.750	0.258
pIgR	3.75^a^	1.42^b^	0.96^b^	2.08^a^	0.33	0.048	0.241	0.003

*Data are presented as mean ± SEM (n = 6 hens /group)

^a-c^ Mean values with different letters differ significantly (p < 0.05)

*SEM* standard error of the mean; *LPS* lipopolysaccharide; *VDR* vitamin D receptor; *RXR* retinoid X receptor; *TLR* Toll-like receptor; *NF-κB* Nuclear factor kB; *TNF-α* tumor necrosis factor; *IFN* interferon; *IL* interleukin; *LYZ* lysozyme; *iNOS* inducible nitric oxide synthase; *pIgR* polyglobulin receptor

## Discussion

Our study showed that VD_3_ deficiency significantly reduced laying performance and egg quality. However, the addition of VD_3_ at 500 IU; 1500 IU; and 3000 IU/kg to the basal diet has been reported to significantly increased egg production, egg weight, and egg quality; consequently, FCR was improved compared with the VD_3_-deficient hens. Moreover, hens fed diets containing 1500 IU and 3000 IU VD_3_/kg of diet had stronger eggshells than birds given 500 IU VD_3_/kg of diet. Consistent with our findings, similar findings on production performance were reported in previous studies [34–36], where production rate, daily egg weight, and feed intake did not differ. Other studies [37, 38] have reported no significant differences in laying performance and egg quality among different level of VD_3_ supplementation in the diet. Thus, our results indicated that suitable supplemental dosage of VD_3_ for middle-phase laying hens is 500 to 3000 IU/kg of diet. Additionally, decreased egg production and egg weight as well as higher mortality rates in the VD_3_-deficient hens were probably because of changes in intestinal microbiota, influences on liver lipid metabolism, and reduced energy utilization efficiency (unpublished data).

25(OH)D, is the major circulating form of vitamin D and is used as indicator of vitamin D status [39]. In our study, the concentration of 25(OH)D and calcium in serum were significantly lower in VD_3_-deficient birds compared with VD_3_-sufficient birds, which was in agreement with previous studies using mouse models [40]. Conversely, the concentrations of 25(OH)D increased in response to dietary VD_3_ supplementation in our study, were accompanied by increases in serum concentrations of calcium, as shown by other researchers’ findings in chickens [41–43]. The role of VD_3_ in calcium and phosphorus metabolism is crucial for its well-documented involvement in bone and eggshell formation in layer hens [44]. Our results have shown that hens fed diets without VD_3_ supplementation showed a significant reduction in egg production as well as poor egg quality, whereas supplementation with VD_3_ significantly improved egg production and eggshell thickness and strength. These changes were accompanied by increased serum calcium and 25(OH)D concentration, implying that VD_3_ deficiency might affect calcium and VD_3_ metabolism, and disturb the process of eggshell formation.

This study further investigated the effects of VD_3_ deficiency and sufficiency on blood hormones and immune function in laying hens challenged with *E. coli* LPS. CORT, as a glucocorticoid, has a potential immune-suppressive and pro-inflammatory cytokine regulatory effect in animals and poultry [45]. Hence, changes in plasma or serum CORT concentration have been commonly used to assess stress and poultry welfare. However, follicle development and ovulation is controlled by complex endocrine interactions that involve the hypothalamic-pituitary-gonadal axis: hypothalamus (gonadotropin releasing hormone), pituitary (FSH, LH), and gonads (E2, PG, T), and many other hormones secreted by these organs [46–48]. Information about the effects of VD deficiency and sufficiency on reproductive hormones is limited. In our study, relative to saline-injected hens, injection with LPS significantly reduced the level of E2, LH, T, and PG in the serum and significantly increased serum CORT concentration, whereas there were no significant effects on PTH and FSH. The elevated CORT and reduced sex steroid hormones in laying hens could be attributed to acute stress caused by LPS. However, VD deficiency results in a significant reduction in FSH and LH levels, and a remarkable increase in E2 and PG regardless of LPS challenge. However, the changes induced by VD_3_ deficiency were completely reversed by VD_3_ supplementation. Furthermore, LH concentration was significantly increased in LPS-challenged and VD_3_-supplemented groups compared to the LPS-challenged and VD_3_-deficient treatment. Increased LH concentration and decreased E2 and PG levels in VD_3_-supplemented groups implied that addition of sufficient VD_3_ could counteract the negative feedback effect of E2 on hypothalamic and pituitary hormone secretion, and positively affect follicle development and ovulation by inhibiting E2 levels. Significant reduced egg production observed in VD_3_-deficient hens might be involved in the lower LH and higher levels of E2 and PG in serum. This finding accounts for the beneficial effects of VD supplementation on hens’ laying performance, possibly related to reproductive hormones.

Calcium homeostasis reflects the balance between fluxes to and from the gut, regulated renal reabsorption, and deposition/mobilization by bone. This process is primarily regulated by the complex interactions of PTH, CT, and vitamin D, as well as with direct exchange with the bone matrix [49]. PTH secreted by the chief cells of the parathyroid glands regulates renal synthesis of 1,25-(OH)_2_-D_3_, increasing the concentration of calcium in the blood. PTH secretion is stimulated by hypocalcemia; its concentration provides an important index of vitamin D/calcium status, whereas CT decreases calcium concentration [50]. In our study, relative to saline-injected hens, injection with LPS significantly reduced serum CT, whereas it had no significant effect on PTH. This finding showed that LPS challenge could increase serum calcium levels by inhibiting CT secretion. Supplementation of VD_3_ resulted in reduced serum CT levels of the birds relative to VD_3_-deficient treatment, which was not in agreement with the finding of Jiang et al. [42]. This change likely reflects increases in serum calcium. However, lower serum PTH content was observed in the VD-supplemented hens, which is consistent with the results of Jiang et al. [42] this change might be helpful to suppress the reabsorption, and deposition and mobilization by bone and help to maintain blood calcium homeostasis in the VD_3_-supplemented diets with normal level of dietary calcium and phosphorus. Increased PTH, however, was observed in VD_3_-deficent hens, resulting in calcium resorption in the kidneys or intestines; this might causes change in serum calcium. Accordingly, these results suggested that reduced eggshell quality in the VD_3_-deficient group was because of effects on PTH and CT concentrations affecting serum calcium levels.

MPO is most abundantly expressed in neutrophil granulocytes; it participates in innate immune defense and plays a key role in the resolution of inflammation [51]. The complement system has been long recognized as a central part of innate immunity in defense against pathogen invasion [52, 53]. In our study, VD_3_ deficiency significantly increased MPO activity, while LPS-injected hens fed with VD_3_-supplemented diet showed a significant reduction in MPO activity compared with saline-injected hens without VD_3_ supplementation. These results showed that VD_3_ deficiency could cause inflammation; however, VD_3_ supplementation not only reversed VD_3_ deficiency–induced inflammatory responses but also alleviated immunological inflammation caused by LPS through reducing MPO activity. In addition, we also observed that serum complement C3, C4, and C5 activity were not affected by VD_3_ supplementation, which is in agreement with the report of Zhang et al. [54] who found that dietary VD_3_ had no effect on serum complement level in fish. However, the higher level of 25(OH)D in human serum will directly accompany increasing C4 concentration but decreasing C3 concentration [55]. The inconsistent results might contribute to different biological species. Our findings indicated that VD_3_ apparently did not activate the complement system.

Natural antibodies are essential components of the innate immune system, and they are present in the body without known antigenic stimulation of B cells [56]. In our study, VD_3_ deficiency promoted serum IgG production, whereas VD_3_ supplementation significantly reversed this change. This is consistent with other findings [57] indicating VD_3_ downregulated T cell–driven IgG production or inhibited antibody production through preventing the proliferation of activated B cells [24, 58, 59]. In hens not injected with LPS, however, VD_3_ supplementation was associated with a significant increase in serum IgM, whereas lower levels of total IgM were observed in hens injected with LPS and fed with sufficient VD_3_. This observation suggested that VD_3_ supplementation could be beneficial in enhancing the innate humoral immunity of hens reared in an unchallenged environment, while counteracting the inflammatory response when confronted by an inappropriate and overly exuberant immune reaction caused by LPS through inhibiting IgM production [60]. Therefore, VD_3_ may be a useful immune regulator in layer production.

In our study, VD_3_ deficiency resulted in a significant increase in the proportion of CD3^+^ T lymphocytes, as well as in a remarkable reduction in the regulatory CD4^+^CD25^+^ T cell population, while these changes were reversed by VD_3_-sufficient supplementation regardless of LPS administration. Similar results have been reported in humans [61, 62], mice [63], and chickens [24]. Vitamin D has been reported to inhibit Th1 and Th17 responses, induce regulatory T cell responses, and control proliferation and helper T cell localization [64, 65]. Regulatory T cells are the subset of CD4^+^ T cells that express FOXP3 protein and the cell surface marker CD25 with anti-inflammatory and immunosuppressive properties by the secretion of potentially inhibitory cytokines IL-10 and TGF-β [66]. Therefore, the results showed that VD_3_ deficiency could activate a T cell–mediated immune response and possibly lead to an inflammatory response with elevated levels of CD3^+^ T cells, whereas VD_3_ supplementation could suppress an inflammatory response by activating regulatory T cells [67] and converting naïve T cells into regulatory T cells and preserve immune homeostasis [9, 13, 68]. In addition, in the absence of LPS challenge, the percentage of CD8^+^ T cells in circulation was not influenced by VD_3_ treatment, whereas in the presence of LPS challenge, LPS induced an increase in the numbers of CD8^+^ T cells. This increase was reversed by supplemental VD_3_. In contrast to our findings, Morris et al. [24] indicated that feeding 25(OH) D increased CD8^+^ cell percentage in the cecal tonsils of pigs. CD8^+^ T cells, the cytotoxic T cells, are effector T lymphocytes, essential in immune protection against intracellular pathogens. Lower CD8^+^ T cell populations observed in VD_3_-sufficient animals has been suggested to have a protective role against LPS-induced immunological stress. Selevaraj et al. [69] found that VD_3_ may inhibit T cell proliferation and the recruitment and activation of T-cells through CXC chemokines at the site of infection and may act as a potential anti-inflammatory agent. Therefore, our results suggested that VD_3_ supplementation suppressed LPS-induced inflammatory response by modulating T cell subset differentiation and T lymphocyte effector function. We speculated this role of VD_3_ is possibly carried out through regulating VDR or stimulating VD-related enzyme expression.

The above results implied that vitamin D deficiency depressed the immune function of chickens. In addition, immunity is also associated with inflammation, which is primarily mediated by inflammatory cytokines in chicken. We further examined the effects of VD_3_ deficiency and sufficiency on serum cytokines and splenic immune-related gene expression in laying hens challenged with *E. coli* LPS. In our study, LPS stimulation upregulated all proinflammatory cytokines (TNF–α, IL-1β, IL-2, IL-4, IL-6, IL-8) in the serum of the laying hens. Vitamin D administration differentially influenced cytokine profiles, with a reduced production of proinflammatory cytokines IL-6, and the ratio of IFN-γ to IL-4 (Th1/Th2). Vitamin D administration showed a contradictory result for Th2 cytokine IL-10 expression, in contrast to the observation of increased IL-10 production in other studies [70]. This finding was in contrast to the vitamin D–deficient group irrespective of LPS. Similar findings relating to VD_3_ and its metabolites inducing pro-inflammatory and anti-inflammatory cytokine production stimulated by bacteria and bacterial PRR exposure were reported by previous authors in human [71, 72], murine [31, 73], porcine [24], and avian systems [21–23, 74]. Boodhoo et al. [74] demonstrated that vitamin D reduces chicken T lymphocyte proliferation as well as the number of IFN-γ producing cells. In addition, it has been previously shown that VD_3_ can modify the balance between the Th1 and Th2 response [70]. The decreased ratio of IFN-γ to IL-4 in the blood of VD-sufficient hens, regardless of LPS injection, may suggest that VD suppresses production of Th1 cytokines and promotes Th2 responses. Furthermore, in clinical settings, a reduction in IFN-γ production may reduce the immunopathology observed in acute or chronic inflammatory diseases induced by infectious agents. Therefore, these results indicated that VD_3_ deficiency is associated with immunological hyperactivity and could cause an inflammatory response. The response could result from enhancing Th1-derived responses and suppressing Th2-driven responses, while VD supplementation dampened the inflammatory response from LPS through suppression of pro-inflammatory Th1 in favor of an anti-inflammatory Th2 phenotype, but without causing immunopathology.

The spleen is an important peripheral immune organ, comprising B cells, T cells, macrophages, and dendritic cells. Splenic cells secrete cytokines following immune stimulation and play an important role in systemic immune function. Toll-like receptor 4 recognizes the LPS of gram-negative bacteria such as *E. coli* and *Salmonella* and stimulates the intracellular cascades to activate nuclear factor-κB. In turn, this stimulation leads to the synthesis of cytokines and other molecules and contributes to the initiation of an inflammatory response. In our study, we observed upregulated expression of splenic TLR4, NF-κB and IL-6 gene mRNA levels in the spleen following LPS challenge. This finding may imply a robust inflammatory response caused by LPS. Meanwhile, significant upregulated expression of RXR-α, LYZ, iNOS, and pIgR genes in the spleen were found in VD_3_-deficient hens, indicating that VD_3_ deficiency could cause inflammation in laying hens. This observation was in accordance with the results reported by previous researchers [75]. Interestingly, in the presence of LPS, VD_3_ supplementation significantly upregulated splenic TLR2, LYZ, and pIgR gene mRNA but downregulated the expression of the NF-κB gene in the spleen relative to single LPS-injected hens. These results suggested that VD_3_ alleviates LPS-induced splenic immunological stress through suppressing the transcription of NF-κB genes and enhancing innate immunity. Previous studies have demonstrated that vitamin D exerts its anti-inflammatory activity response to LPS stimulation through decreasing gene expression associated with inflammation [22, 71] as well as by blocking NF-κB activation [31, 71, 76, 77]. Thus, the observed effects of vitamin D on the inhibition of LPS-induced NF-κB activation in hens might be because of the anti-inflammatory effect of VD_3_ [78–80]. Although previous studies having demonstrated that LPS downregulates VDR expression in keratinocytes [81], vitamin D and its metabolites could alter the expression of different genes for immunoregulation by binding to VDR [82, 83], VDR gene expression was not influenced by LPS or VD_3_ treatment in our study and needs to be investigated further.

## Conclusion

Our study showed that VD_3_ deficiency caused decreased egg production, poor eggshell quality, and inflammatory responses in laying hens. Dietary VD_3_-sufficient supplementation could improve egg production and egg quality. Supplementation could also repress immune inflammatory responses caused by LPS administration or VD_3_ deficiency by affecting reproductive hormone secretion and regulating the NF-κB signaling pathway. Thus, VD_3_ supplementation could be beneficial to protect layer hens in both preventing immunological stress and VD_3_ deficiency. We plan to further investigate the potential mechanisms of action related to the anti-inflammatory effect of VD_3_ in chickens.

## Abbreviations

1,25-(OH)_2_D_3_: 1α,25-dihydroxycholecalciferol
25(OH)D: 25-hydroxycholecalciferol
C3: Complement 3
C4: Complement 4
C5: Complement 5
CORT: Cortisone
CT: Calcitonin
E2: Estradiol
FCR: Feed conversion rate
FSH: Follicle stimulating hormone
IFN: Interferon
IgG: Immunoglobulins G
IgM: Immunoglobulins M
IL: Interleukin
iNOS: Inducible nitric oxide synthase
LH: Luteinizing hormone
LPS: Lipopolysaccharide
LYZ: Lysozyme
MPO: Myeloperoxidase
NF-kB: Nuclear factor-kB
PG: Progesterone
pIgR: Polyglobulin receptor
PTH: Parathyroid hormone
RXR: Retinoid X receptor
T: Testosterone
TLR: Toll-like receptor
TNF-a: Tumor necrosis factor
VD_3_: Vitamin D_3_
VDR: Vitamin D receptor
